# Novel insights into athlete physical recovery concerning lactate metabolism, lactate clearance and fatigue monitoring: A comprehensive review

**DOI:** 10.3389/fphys.2025.1459717

**Published:** 2025-03-25

**Authors:** Tianhao Huang, Zeyu Liang, Ke Wang, Xiangjun Miao, Lei Zheng

**Affiliations:** China Basketball College, Beijing Sport University, Beijing, China

**Keywords:** athlete, physical recovery, lactate metabolism, lactate clearance, fatigue monitoring

## Abstract

Lactate accumulation will appear in athlete skeletal muscle after intense exercise. If the high lactate level maintains, athletes will sustain fatigue and athletic capacity decline due to internal environment and normal metabolism disruption. In order to enhance athlete physical recovery and exercise performance in high-intensity sport events, it is of great significance to explore the scientific intervention procedures based on quicker lactate clearance in skeletal muscle and blood after exercise. This article collects classic and novel literature in terms of lactate metabolism, lactate clearance and fatigue monitoring during exercise by searching PubMed database and then summarizes comprehensive insights into athlete physical recovery with corresponding figures and charts. We introduce the generation and transformation process of lactate, lactate clearance pathways and the fatigue monitoring methods for athletes in detail. The lactate clearance pathways involve biochemical pathways (oxygen inhalation, amino acids supplement, targeting free radical, alkaline reserve, targeting vasomotion, ribose supplement), physical activities (exercise-mediated activities, non-exercise activities) and training methods (interval training, altitude training) to accelerate lactate metabolism. The biochemical factors for monitoring athletic fatigue level involve blood, urine, sweat, saliva and exhaled gas. We hope this review can offer some significant and scientific assistance for athlete recovering after exercise and improving sport achievements based on quicker lactate clearance.

## 1 Introduction

After long-time and high-intensity training and competition, the body of athletes will show fatigue due to excess carbohydrate consumption and oxidation function decline, followed by metabolic imbalance, cell membrane oxidative damage, serum lactate accumulation (Von Duvilard et al., 2004; Kaviani et al., 2020; Kruk et al., 2021). When the acidic metabolites generated during exercise reduce the acidity of body fluids to the threshold, the blood pH will decrease and the electrolyte balance will break, which will greatly impair the exercise performance of athletes (Kamińska et al., 2020; Zhu et al., 2020). At present, there is little effective ways to directly eliminate sport fatigue, and high-intensity training and competition require more effective physical recovery for athletes. Thus, it is necessary and significant for developing scientific recovery procedures with fewer side effects and no doping ingredients in athlete physical recovery.

During exercise, lactate is generated from anaerobic glycolysis in skeletal muscle and delivered to the body through blood circulation, taking a certain time to be metabolically cleared (Hall et al., 2016). Normally, lactate will be eliminated in a period of time after exercise. But for athletes, long-term intense exercise will induce the imbalance between lactate generation and clearance, lactate accumulation will eventually cause fatigue and prolong athlete physical recovery (Lee et al., 2023). Hence, serum lactate content is an important index to measure and judge the fatigue of athletes. To monitor the blood lactate level of athletes after exercise, and develop scientific recovery procedures for lactate clearance according to the principle of lactate metabolism will help to improve the physical recovery and exercise performance of athletes.

In this study, we search and download the classic and novel literature concerning lactate metabolism, lactate clearance and fatigue monitoring during exercise from PubMed database. The comprehensive understandings of athlete physical recovery have been summarized and the analysis results are shown in corresponding figures and charts. As follow, we introduce the generation and transformation process of lactate, lactate clearance pathways and the biochemical factors of monitoring fatigue for athletes in detail, hoping to provide a certain theoretic and practical basis for exploring the scientific intervention procedures based on quicker lactate clearance after exercise.

## 2 Lactate metabolism: Lactate generation and transformation in athletes after exercise

**FIGURE 1:**
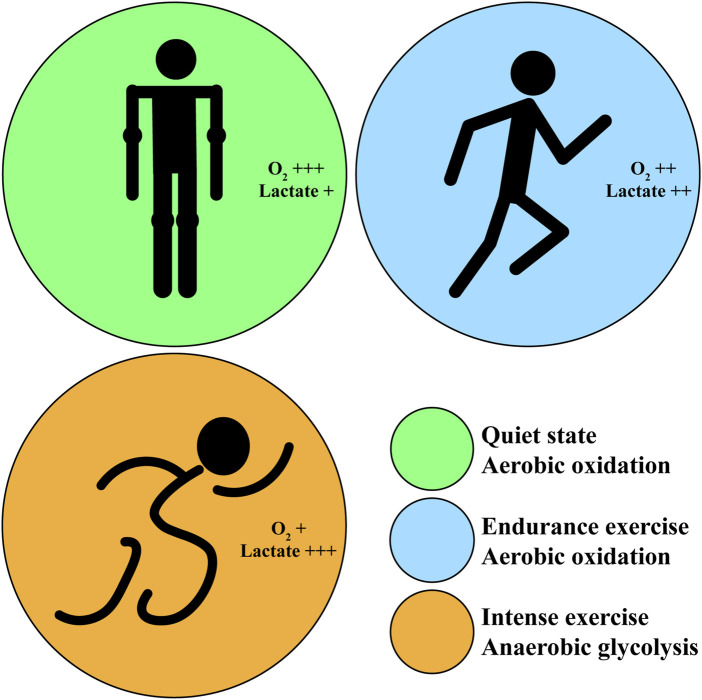
The oxygen consumption and lactate generation corresponding to different exercise intensity. Green circle: in quiet state, the body proceeds aerobic oxidation and generates little lactate with sufficient oxygen. Blue circle: in endurance exercise, the body proceeds aerobic oxidation and generates more lactate with decreasing oxygen; athletes feel fatigue after exercise. Orange circle: in intense exercise, the body proceeds anaerobic glycolysis and generates excess lactate with little oxygen; athletes feel very fatigue after exercise.

In human body, the energy supply pathways of carbohydrate metabolism mainly include aerobic oxidation and anaerobic glycolysis in skeletal muscle, which play an essential role in exercise maintaining. In different exercise patterns, the energy supply pathways of carbohydrate metabolism is different. The aerobic oxidation of carbohydrate is mainly applied in long-term endurance exercise or quiet state, while anaerobic glycolysis is mainly applied in short-time intense exercise (Figure 1).

**FIGURE 2:**
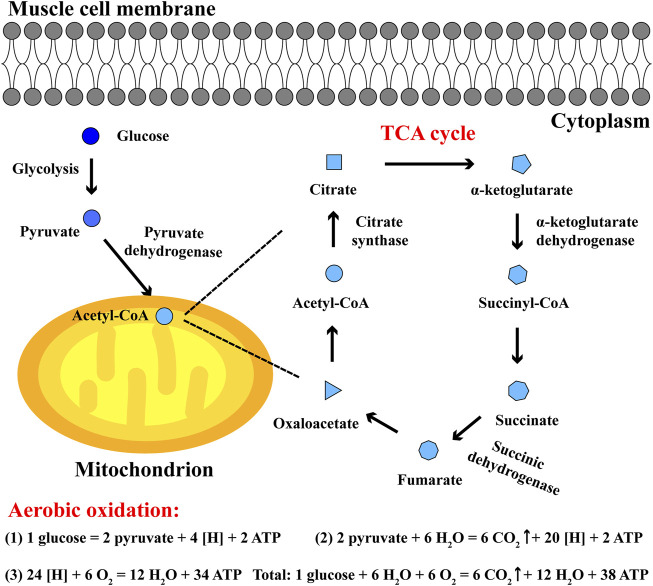
The aerobic oxidation and TCA cycle in skeletal muscle cells. In cytoplasm, glucose converts to pyruvate; in mitochondria, pyruvate converts to acetyl-CoA and enters TCA cycle. In aerobic oxidation, one glucose eventually generates 6 CO_2_, 12 H_2_O and 38 ATP during endurance exercise.

The main process of carbohydrate aerobic oxidation involves: in cytoplasm, glucose converts to pyruvate; in mitochondria, pyruvate converts to acetyl-CoA and enters tricarboxylic acid cycle (TCA) with oxaloacetate; ATP is generated from electron transfer chain and substrate level phosphorylation, accompanied by CO_2_ and H_2_O (Tran et al., 2019; Hsu et al., 2019). TCA cycle is a common pathway of energy metabolism and transformation in the body, and plays a key role in carbohydrate aerobic oxidation. Among them, the main catalytic enzymes of TCA cycle include citrate synthase, α-ketoglutarate dehydrogenase, succinic dehydrogenase and so on, which are highly expressed in skeletal muscle tissues. Oxaloacetate and acetyl-CoA converting into citrate requires the catalysis of citrate synthase, the oxidative decarboxylation of α-ketoglutarate converting into succinyl-CoA and CO_2_ requires α-ketoglutarate dehydrogenase, and succinic dehydrogenase plays a regulatory role in the oxidation of succinate converting into fumarate (Ren et al., 2020; Hansen and Gibson, 2022; Balnis et al., 2021). Their activities can be generally applied as an indicator to characterize the progress of TCA cycle and the balance between aerobic oxidation and anaerobic glycolysis during exercise (Figure 2).

**FIGURE 3:**
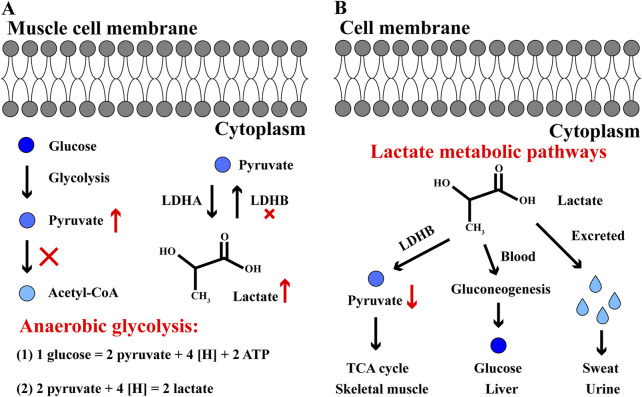
The anaerobic glycolysis and lactate metabolic pathways in skeletal muscle cells **(A)** In cytoplasm, glucose converts to pyruvate, but pyruvate cannot convert to acetyl-CoA and enter TCA cycle. In anaerobic glycolysis, one glucose eventually generates two lactate and 2 ATP during intense exercise. **(B)** Three pathways of lactate metabolism. Firstly, lactate converts to pyruvate and enter TCA cycle in skeletal muscle; secondly, lactate converts to glucose through gluconeogenesis in liver; thirdly, lactate is excreted in sweat and urine. The red and up arrow indicates increasing effect, the red and down arrow indicates reducing effect, the red cross indicates inhibitory effect.

The chemical formula of lactate is C_3_H_6_O_3_ and its molecular weight is 90.08. In aqueous solution, the carboxyl group releases a proton to produce the lactate ion CH_3_CH(OH)COO^−^. Generally, lactate level rises when tissue energy cannot be satisfied through aerobic oxidation. Skeletal muscle cannot obtain enough oxygen, or it cannot process oxygen quickly enough. In this case, pyruvate dehydrogenase is unable to convert pyruvate into acetyl-CoA in time, and pyruvate begins to accumulate. In mammals, there are three main subtypes of lactate dehydrogenase (LDH): LDHA, LDHB and LDHC (Deme and Telekes, 2017). During exercise, LDH mainly regulates the metabolic balance of lactate and pyruvate in skeletal muscle, where LDHA and LDHB are abundant. If pyruvate cannot be completely metabolized by aerobic oxidation, LDHA will mediate the transformation of pyruvate into lactate; in contrast, LDHB mediates the transformation of lactate into pyruvate when pyruvate is significantly reduced (Figure 3A) (Read et al., 2001; Gupta, 2012). During high-intensity exercise, LDHA plays a dominant role in order to improve the anaerobic glycolysis efficiency of skeletal muscle.

The lactate generated by skeletal muscle during exercise mainly metabolizes into three pathways after exercise: firstly, in skeletal muscle, lactate oxidizes into CO_2_ and H_2_O; secondly, lactate enters into liver with blood circulation and converts to glucose/glycogen through gluconeogenesis; thirdly, it is excreted in sweat and urine (Figure 3B) (Hui et al., 2017; Chen et al., 2021; Wang et al., 2022). Among these three metabolic pathways, oxidation is the main destination for lactate metabolism after exercise. With LDH catalytic reaction, lactate is dehydrogenated and oxidized to pyruvate, which is completely oxidized and decomposed through TCA cycle (Park et al., 2022). Thus, the serum LDH content and activity obviously increase and the maximum appear after exercise. Meanwhile, lactate accumulation after exercise will increase the blood lactate level, and in the long run, pathologically elevated lactate levels can induce protein lactation in the body, eventually improve the development of several diseases such as sepsis, tumor, Alzheimer’s disease (Huang et al., 2023). Hence, it is necessary and significant for developing scientific recovery procedures in athlete physical recovery based on quicker lactate clearance.

## 3 Lactate clearance: Pathways to clear lactate in athletes after exercise

According to the lactate threshold, serum lactate in 2–4 mM represents aerobic metabolism and low-intensity exercise; 4–12 mM represents aerobic metabolism transforming to anaerobic metabolism and moderate exercise; over 12 mM represents anaerobic metabolism and high-intensity exercise (Sliwowski et al., 2013). The serum lactate level can be applied to monitor the exercise intensity and fatigue level of athletes. And the key to effectively clear lactate is to select proper methods according to the understanding of the essence of lactate metabolism. In this section, we introduce scientific recovery procedures to clear lactate in athletes after exercise, including biochemical pathways, physical activities and training methods to accelerate lactate metabolism (Table 1).

**TABLE 1:** The pathways for lactate clearance.

Pathways	Target	Treatment	Ref.
Biochemical pathways			
Oxygen Inhalation	Hypoxia	Uptake of high concentration O_2_	(22,23)
Amino Acids Supplement	TCA cycle	Aspartic acid and glutamic acid	(24–26)
Targeting Free Radical	Oxidative stress	Polyphenols and vitamins	(27–30)
Alkaline Reserve	Blood acidity	Alkaline food and alkaline drinks	(31–34)
Targeting Vasomotion	Blood obstruction	Herbal medicine	(35)
Ribose Supplement	Energy supply mode	Ribose	(36–38)
Physical activities	Muscle tension	Exercise-mediated activities Non-exercise activities	(39–51)
	Blood circulation		
Training	Lactate threshold	Interval Training	(52–58)
		Altitude Training	

### 3.1 Biochemical pathways to accelerate lactate metabolism

#### 3.1.1 Oxygen inhalation

The body still maintains high metabolic level and high oxygen consumption after exercise, suffering oxygen starvation for a period of time. Then lactate cannot convert into CO_2_ and H_2_O *via* oxygenolysis as soon. The uptake of high concentration oxygen can quickly elevate the blood oxygen saturation to a higher level, which accelerates lactate oxygenolysis and restores internal pH; Meanwhile the improvement of hypoxic environment can enhance the antioxidant system to eliminate peroxide and accelerate physical recovery (Hauser et al., 2014; Xiao and Li, 2006).

#### 3.1.2 Amino acids supplement

Oxygenolysis is the main metabolic pathway of lactate after exercise, including converting to pyruvate and TCA cycle. Pyruvate is converted to acetyl-CoA with pyruvate dehydrogenase catalytic reaction, and acetyl-CoA plus oxaloacetate generate citrate to initiate TCA cycle, thereby the oxidation rate of lactate is associated with the TCA cycle initiator oxaloacetate (Figure 2) (Chhimpa et al., 2023). Meanwhile aspartic acid and glutamic acid can be converted into oxaloacetate (Holeček, 2023; Hertz and Hertz, 2003). Thus supplement with aspartic acid and glutamic acid after exercise will promote the synthesis of oxaloacetate and enhance the oxidation of lactate in TCA cycle, so as to quickly clear lactate in muscle and accelerate the recovery of blood lactate level.

#### 3.1.3 Targeting free radical

High-intensity exercise dramatically increases free radical generation in skeletal muscle due to the aggravated oxidative stress, and the free radical-mediated cellular damage will induce aerobic and anaerobic metabolism disorder which impairs the lactate clearance after exercise to a certain extent (Parker et al., 2014). Thus, targeting free radical is an important strategy for athletes decreasing blood lactate level and recovering after exercise. Some studies indicated that the polyphenols in fruits and tea and vitamins showed great antioxidant capacity to protect against exercise-induced free radical, consequently improving lactate clearance and sport performance (Parenteau et al., 2023; Zhao et al., 2022; Peng et al., 2009). Hence the supplement of polyphenols and vitamins are recommended for athletes in daily training.

#### 3.1.4 Alkaline reserve

There are buffer solutions such as bicarbonate and hydrophosphate in the blood to maintain body acid-base balance, among which bicarbonate buffer pair has a strong buffer capacity, and the physically dissolved CO_2_ in the blood can maintain the dynamic stability of H_2_CO_3_ (McKay et al., 2020; Pilch et al., 2020). When lactate enters the blood from skeletal muscle, it combines with carbonate to generate H_2_CO_3_, which can antagonize the changes in blood acidity caused by lactate accumulation. However, the self-reserve of alkaline in athletes cannot afford to neutralize excess lactate. Before training and competition, athletes are recommended to properly supplement alkaline food and alkaline drinks, so that the improvement of alkaline electrolyte storage can enhance the athletic ability to neutralize blood lactate, delay the decline of exercise performance and reduce exercise fatigue (Baranauskas et al., 2020; Newbury et al., 2021).

#### 3.1.5 Targeting vasomotion

During exercise, skeletal muscle contraction causes mechanical compression of vascular endothelial cells, and the reduced blood flow aggravates hypoxia process. After exercise, the contraction of skeletal muscle relieve slowly and the breathing frequency of athletes reduces, leading to the slow vasomotion and lactate oxygenolysis. Recent years, herbal medicine like ginkgo biloba extract has been applied to promote endothelial cell relaxation, increase erythrocyte deformability and reduce blood viscosity, so that the vasomotion after exercise liberates blood flow and enhance lactate oxygenolysis with normal oxygen saturation (Kim et al., 2010).

#### 3.1.6 Ribose supplement

In addition to the glycolic and oxidative pathways, pentose phosphate pathway is another complex energy supply mode of carbohydrate oxidation and ATP production (Icard and Lincet, 2012; Ghergurovich et al., 2021). In high-intensity exercise, if we can enhance the sport effects of pentose phosphate pathway supplying ATP in a synergistic manner, the elevating proportion of prophosphate energy supply and the decline of glycolysis will reduce lactate generation and improve athletic lactate metabolism. A randomized controlled trial showed that ribose supplement is recommended to effectively clear lactate after exercise based on alternative energy supply mode (Cao et al., 2020).

### 3.2 Physical activities to accelerate lactate metabolism

The traditional idea of sport training believes that intensity is the core factor to improve athletic ability, but excess exercise will cause sport injuries. With the change of the focus of modern sport training concept, more scholars and coaches realize that the improvement of athlete competitive level depends more on the accumulation of sport effects. Besides exercise intensity, athletes should pay attention to fatigue recovery in order to obtain better sport achievement. With the deeper understanding of the field of exercise fatigue recovery, the importance of controlling exercise intensity and recovery to training effect has become increasingly prominent. Therefore, exercise recovery is an essential way to improve training effect and sport performance, and special emphasis is placed on the positive effect of physical activities accelerating lactate metabolism.

After competition or training, the body of athletes still maintain in a high metabolic level, the sudden stop of exercise will reduce blood flow and oxygen, blocking the blood lactate dispersion with blood circulation. The lactate accumulation is not conducive to lactate clearance. Nowadays, in terms of lactate clearance, physical activities of exercise recovery has evolved into many forms, which can be divided into two broad categories: exercise-mediated activities and non-exercise activities. Both of them play a vital role in relieving muscle tension, improving blood circulation, replenishing oxygen, regulating endocrine system and accelerating lactate metabolism. The exercise-mediated activities involve: jog (Losnegard et al., 2015), stretch (Yui et al., 2021), bicycle ergometer (De Ridder et al., 2022) the non-exercise activities involve: sleep (Rempe and Wisor, 2015), muscle massage (Ferreira et al., 2023), foam roller (Adamczyk et al., 2020), cold and warm immersion (Adamczyk et al., 2016; Sautillet et al., 2024), acupuncture (Rosa et al., 2023), foot reflexology (Cai et al., 2022), light irradiation (Baroni et al., 2010), magnetic treatment (Lefaucheur et al., 2017), electric nerve stimulation (Neric et al., 2009) and so on.

### 3.3 Training to raise lactate threshold

With the increase of exercise intensity, the blood lactate concentration of athletes will sharply rise at a certain node, called lactate threshold, and the corresponding exercise intensity is lactate threshold intensity (Faude et al., 2009). The emergence of lactate threshold represents the energy supply of athletes transforming from aerobic oxidation to anaerobic glycolysis. In terms of innate factor and acquired training, the changes of blood lactate level and lactate threshold of athletes are different, which can objectively reflect the exercise intensity, aerobic and anaerobic metabolic capacity of athletes (Löllgen and Leyk, 2018). Thus lactate threshold is applied as a reference by coaches to schedule training and control training load, meanwhile applied as a training method to improve athlete endurance, including interval training and altitude training.

#### 3.3.1 Interval training

As a grouped training, interval training contains exercise intensity from low to high and recovery time, which emphasizes multiple groups of repetition and is characterized by short time and high energy consumption (Seiler et al., 2013; Haugen et al., 2021). The recovery time among training can improve the lactate release from skeletal muscle to blood and lactate clearance, compared with continuous training, interval training allows athletes experience more intense training by synergizing the recovery time and training intensity, which can effectively improve the endurance and competitive level of athletes. An animal study showed that after running intervention of interval training, the endurance performance of rats was improved more than continuous training, with the enhancing AMPK-PGC-1α signaling pathway, a key factor of mitochondria metabolism regulation (Pengam et al., 2023).

#### 3.3.2 Altitude training

In the altitude with low oxygen, athletes suffer double hypoxia stimulation during high-intensity training, so that the energy supply of anaerobic glycolysis of skeletal muscle stands greater test than that in the plain with rich oxygen, which improves the generation, tolerance, transformation of lactate and the anaerobic metabolic capacity of athletes (Millet et al., 2010). In the training of same intensity, the level of blood lactate after altitude training is higher than that of plain training, indicating that the elevated anaerobic glycolysis promotes the generation and transformation of blood lactate. Hence, altitude training can elevate lactate threshold and the ability of oxidative clearance of blood lactate, showing a positive enhancing effect on the physiological performance and athletic capacity of athletes. A current observational study found that moderate altitude significantly increased the lactate threshold velocity and heart rate and upper body muscle mass of elite Chinese cross-country skiers after over 4 weeks of training (Yu et al., 2022).

## 4 Fatigue monitoring: Biochemical factors for monitoring athlete fatigue level

Sports load is increasing with the continuous elevation of modern athletic level. Moderate exercise fatigue with scientific recovery procedures can promote the exercise performance of athletes, but excessive fatigue is unfavorable to improve athletic capacity, causes sport injuries and damages athlete physical health. Thus it is of great theoretical and practical significance to accurately monitor sport fatigue and define fatigue degree for making scientific training plan, improving training effect and avoiding injuries caused by excess fatigue. In this section, we introduce several biochemical factors and the detection methods for monitoring athletic fatigue level, including blood, urine, sweat, saliva and exhaled gas (Table 2).

**TABLE 2:** The biochemical factors and methods for monitoring athletic fatigue.

Biochemical factors	Biochemical markers	Methods	Ref.
Blood	Lactate	Colormetric	(63)
	LDH	Sandwich-ELISA	(64)
	Hemoglobin	Sandwich-ELISA, a smartphone-based testing platform	(66)
	ROS	Fluorescent probe	(67)
Urine	Urine protein	Coomassie brilliant blue, biuret and benzethonium chloride	(69–71)
	Creatinine	Colormetric, mass spectrometry	(72,73)
Sweat	pH	Colormetric	(74)
	Lactate	Colormetric, a wearable chemical biosensing system	(63, 75)
Saliva	Immunoglobulin	Sandwich-ELISA	(78)
	Testosterone	Competitive-ELISA	(79)
Exhaled gas	Glucose, amino acid, urea	Mass spectrometry	(80)

### 4.1 Blood

The monitoring of blood components can provide abundant information reflecting the changes of athlete body functions, their biochemical markers such as lactate, hemoglobin, testosterone, cortisol and urea have been widely applied in the human movement science investigation (Krishnan et al., 2022; Kloner et al., 2016; Powell et al., 2013; Batiha et al., 2022). Intense exercise consumes excess energy, so that skeletal muscle utilizes lactate metabolism to maintain the energy supply in a short time. Large amount of lactate is generated from LDH oxidation and released into the blood circulation, making athletes feel more fatigue. Thus the lactate and LDH levels in the blood after exercise are valuable indicators to monitor athletic fatigue, which can be measured by common methods of colorimetric (Meng et al., 2023) and sandwich-enzyme-linked immunosorbent assay (ELISA) kits (Li et al., 2020). After high-intensity exercise, the aggravated oxidative stress in the skeletal muscle of athletes increases the free radical level to impair erythrocyte membrane, leading to the release of intracellular components in blood like hemoglobin, metabolism disorder and endurance loss (Higgins et al., 2020). Thus measuring hemoglobin release and reactive oxygen species (ROS) level can reflect the extent of athletic fatigue, with sandwich-ELISA (Dönmez Türkmen et al., 2022) and fluorescent probe (Xiao et al., 2023) methods, respectively. Fu Q et al. develops a portable and rapid smartphone-based hemoglobin point-of-care testing platform with azide-hemiglobin method which might be employed for athletes monitoring hemoglobin level in the blood after exercise, especially the advantages of low price and field test in playground (Fu et al., 2022). However, blood drawing will cause minor trauma of athletes and it take time to separate serum from blood samples. In recent years, non-invasive detection has been continuously improved in urine, sweat, saliva and exhaled gas, showing important application value in monitoring athletic fatigue.

### 4.2 Urine

Athletes consume vast nutrients during high-intensity exercise, and the dramatic changes in urine composition can be employed to monitor the degree of fatigue of athletes. For urine protein, the secretion of epinephrine and norepinephrine during exercise will lead to its elevation, and the common detection methods include Coomassie brilliant blue (colormetric), biuret (colormetric) and benzethonium chloride (turbidimetric) (Aoki et al., 2023; Hokazono et al., 2021; Özcan et al., 2024). For creatinine, the metabolite of creatine and indicator of muscle fatigue, its metabolism can be measured by colormetric assay kit (Zhang et al., 2021) and mass spectrometry (Niesser et al., 2012).

### 4.3 Sweat

Perspiration is a manifestation of exercise metabolism and carries plentiful metabolic messages. Measuring the sweat pH by colormetric reagents can indirectly reflect the dehydration of athletes and the degree of athletic fatigue (Hamouti et al., 2011). Lactate is another important indicator of exercise fatigue in sweat and the detection method is the same with blood lactate (colormetric) (Meng et al., 2023). Using flexible substrate and electrode amperometric lactate biosensor, Imani S et al. invents a wearable chemical biosensing system to monitor lactate level on the skin, which might be employed for athletes monitoring sweat lactate level in real time during exercise (Imani et al., 2016).

### 4.4 Saliva

During intense exercise, the decline of adrenoreceptor of athlete salivary glands reduces the salivary immunoglobulin level (sIgA) (Matsubara et al., 2010). Testosterone is a steroid hormone that promotes protein synthesis to enhance muscle strength and erythrocyte growth, closely related to athletic capacity and fatigue (Heather, 2022). The salivary immunoglobulin and testosterone levels are important indicators of athletic function monitoring, their dynamic changes can be measured by sandwich- (Paixão et al., 2021) and competitive-ELISA (Xie et al., 2021), respectively.

### 4.5 Exhaled gas

Using mass spectrometry, more compounds have been identified in exhaled gas, which provides a non-invasive and fast check to monitor athletic fatigue. The exhaled gas reflects athletic cardio-pulmonary function, the glucose, amino acid, urea and other exercise metabolites can be employed to monitor athletic fatigue with mass spectrometry (Chen et al., 2007).

## 5 Discussion

The improvement of athletic level is the result of moderate fatigue caused by training and the body function adapting to new level after recovery. Thus fatigue and recovery are essential parts for athletes training and competition. However, if training overloads and the body can not recover in time, fatigue will gradually accumulate. When the limit of body tolerance is exceeded, it will cause great harm to athletic health. Therefore, the elimination and real-time monitoring of fatigue after exercise have important theoretical and practical significance to arrange scientific training plans, improve training effects, create excellent score, and avoid injuries caused by excess fatigue. For this purpose, we introduce and summarize the generation and transformation process of lactate metabolism, the biochemical pathways, physical activities and training methods to clear lactate, the biochemical factors for monitoring athlete fatigue level in detail in this study.

The lactate accumulation caused by intense exercise will reduce the pH of body fluid to block energy supply by glycometabolism, suppress the sensitivity of muscle fibers to calcium ions and muscle contraction, thereby the impair internal environment and disrupt normal metabolism generate fatigue and damage athletic capacity for athlete body (Cairns, 2006). Hence, the quicker lactate clearance in muscle and blood after exercise is beneficial to eliminate fatigue and enhance exercise performance in high-intensity sport events. In this study, we introduce some scientific pathways without doping violations to clear lactate in athletes after exercise, including biochemical pathways to accelerate lactate metabolism, physical activities to accelerate lactate metabolism and training to raise lactate threshold (Table 1). Particularly, the biochemical pathways to accelerate lactate metabolism involve oxygen inhalation, amino acids supplement, targeting free radical, alkaline reserve, targeting vasomotion and ribose supplement, concerning exercise-mediated hypoxia, main lactate metabolic pathway (aerobic metabolism/TCA cycle), exercise-mediated oxidative stress, lactate-mediated blood acidity, muscle tension-mediated blood obstruction and other energy supply mode instead of anaerobic glycolysis. There are many factors affecting the lactate clearance, including gender and age of athletes, self-athletic ability, sport event features, exercise load and environment, lactate clearance methods and time. A correct understanding of the factors affecting the lactate clearance and a targeted selection of lactate clearance methods are the important prerequisite for the rapid lactate clearance after exercise. Also, the nutrients and medicines applied to clear lactate should be accessible, portable and nontoxic for athletes training and competition. However, only studying the micro-changes of athletic metabolism can effectively clear lactate. Thus, multi-omics analyses such as transcriptome, proteome and metabolome are recommended for further exploring the micro-changes and molecular mechanism of athletic metabolism during exercise and physical recovery based on lactate clearance. Meanwhile, the applicability of each lactate clearance pathway and corresponding sport events necessitates sufficient tests for verification.

According to the different assessment criteria, the methods of exercise fatigue monitoring are mainly involved subjective evaluation, physiological monitoring and biochemical monitoring. The subjective evaluation is a convenient and non-invasive method that athletes fill in sport science questionnaires according to their training conditions, but the results are not objective and accurate, thereby serving as an auxiliary method for fatigue monitoring (Royer et al., 2023). The physiological monitoring can be used to evaluate the athletic status by providing objective data of dynamic changes in brain cell current, heart rate, blood pressure, muscle tension, body weight and other physiological indexes, but the results are interfered by individual differences, low accuracy and other various factors (Burkow-Heikkinen, 2011; Saw et al., 2016; Nocera et al., 2023; Lundstrom et al., 2023). Biochemical monitoring for exercise fatigue has been developed rapidly in recent years, since exercise is closely related to the material and energy metabolism of athletic body, such as carbohydrate, protein, lipid metabolism, and the metabolites are eliminated through circulatory system. The biochemical monitoring methods for athlete fatigue we introduce are based on the metabolites in blood, urine, sweat, saliva and exhaled gas after exercise, indicating the athlete metabolic status. Biochemical monitoring is advanced in providing objective results in athletic rapid, field and non-invasive detection, showing a good application prospect in monitoring athletic fatigue and elevating sport performance. Among them, athletic fatigue is induced and characterized by lactate accumulation, so it’s a key concern to monitor lactate level after exercise. The data from biochemical monitoring will help to monitor lactate level in time after exercise, and effectively clear lactate and elevate sport performance. However, the proper methods applied to monitor lactate level depend on the sport event features, as well as reagents and instruments in lab. And the sensitivity, accuracy and repeatability of monitoring methods in corresponding sport events also necessitate sufficient tests for verification.

## 6 Conclusion

Collectively, our review summarizes the generation and transformation process of lactate metabolism, the biochemical pathways, physical activities and training methods to clear lactate, the biochemical factors for monitoring athlete fatigue level in detail from literature, which shows positive significance in improving lactate clearance of sport players, and provides a certain theoretic and practical basis for guiding sport practice. We will cooperate with the athlete teams of Beijing Sport University and conduct sufficient tests in the corresponding sport events to verify the lactate clearance pathways and fatigue monitoring methods we introduce in this study, in order to offer some help for improving training effects, physical recovery and sport performance of athletes.
